# Association between intrarenal venous flow from Doppler ultrasonography and acute kidney injury in patients with sepsis in critical care: a prospective, exploratory observational study

**DOI:** 10.1186/s13054-023-04557-9

**Published:** 2023-07-10

**Authors:** Kenichiro Fujii, Izumi Nakayama, Junichi Izawa, Noriko Iida, Yoshihiro Seo, Masayoshi Yamamoto, Norimichi Uenishi, Teruhiko Terasawa, Mitsunaga Iwata

**Affiliations:** 1 Department of Emergency and General Internal Medicine, Fujita Health University School of Medicine, 1-98 Dengakugakubo, Kutsukakecho, Toyoake, Aichi 470-1192 Japan; 2 Division of Intensive Care Medicine, Department of Internal Medicine, Okinawa Prefectural Chubu Hospital, Uruma, Japan; 3 Department of Public Health, School of Medicine, Yokohama City University, Yokohama, Japan; 4 Department of Health Data Science, Graduate School of Data Science, Yokohama City University, Yokohama, Japan; 5 Department of Preventive Services, Kyoto University School of Public Health, Kyoto, Japan; 6 Clinical Laboratory, University of Tsukuba Hospital, Tsukuba, Japan; 7 Department of Cardiology, Nagoya City University Graduate School of Medical Sciences, Nagoya, Japan; 8 Cardiovascular Division, Faculty of Medicine, University of Tsukuba, Tsukuba, Japan; 9 Department of Emergency and General Internal Medicine, Fujita Health University Hospital, Toyoake, Japan

**Keywords:** Ultrasonography, Renal congestion, Intrarenal venous flow, Acute kidney injury, Sepsis

## Abstract

**Background:**

Intrarenal venous flow (IRVF) patterns assessed using Doppler renal ultrasonography are real-time bedside visualizations of renal vein hemodynamics. Although this technique has the potential to detect renal congestion during sepsis resuscitation, there have been few studies on this method. We aimed to examine the relationship between IRVF patterns, clinical parameters, and outcomes in critically ill adult patients with sepsis. We hypothesized that discontinuous IRVF was associated with elevated central venous pressure (CVP) and subsequent acute kidney injury (AKI) or death.

**Methods:**

We conducted a prospective observational study in two tertiary-care hospitals, enrolling adult patients with sepsis who stayed in the intensive care unit for at least 24 h, had central venous catheters placed, and received invasive mechanical ventilation. Renal ultrasonography was performed at a single time point at the bedside after sepsis resuscitation, and IRVF patterns (discontinuous vs. continuous) were confirmed by a blinded assessor. The primary outcome was CVP obtained at the time of renal ultrasonography. We also repeatedly assessed a composite of Kidney Disease Improving Global Outcomes of Stage 3 AKI or death over the course of a week as a secondary outcome. The association of IRVF patterns with CVP was examined using Student's *t*-test (primary analysis) and that with composite outcomes was assessed using a generalized estimating equation analysis, to account for intra-individual correlations. A sample size of 32 was set in order to detect a 5-mmHg difference in CVP between IRVF patterns.

**Results:**

Of the 38 patients who met the eligibility criteria, 22 (57.9%) showed discontinuous IRVF patterns that suggested blunted renal venous flow. IRVF patterns were not associated with CVP (discontinuous flow group: mean 9.24 cm H_2_O [standard deviation: 3.19], continuous flow group: 10.65 cm H_2_O [standard deviation: 2.53], *p* = 0.154). By contrast, the composite outcome incidence was significantly higher in the discontinuous IRVF pattern group (odds ratio: 9.67; 95% confidence interval: 2.13–44.03, *p* = 0.003).

**Conclusions:**

IRVF patterns were not associated with CVP but were associated with subsequent AKI in critically ill adult patients with sepsis. IRVF may be useful for capturing renal congestion at the bedside that is related to clinical patient outcomes.

**Graphical abstract:**

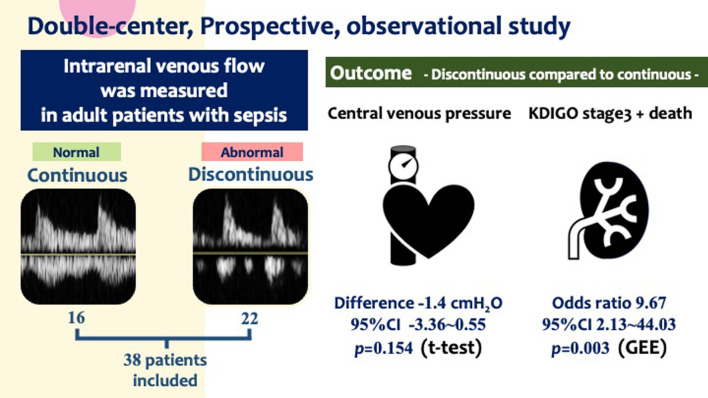

**Supplementary Information:**

The online version contains supplementary material available at 10.1186/s13054-023-04557-9.

## Background

Fluid overload is common during sepsis resuscitation and is associated with acute kidney injury (AKI) and mortality [1–3]. Recent findings have suggested that renal congestion (elevated renal interstitial pressure and impaired renal venous return) is potential mechanisms for AKI after fluid overload [4]. However, other than conventional surrogates for systemic congestion (e.g., central venous pressure (CVP), cumulative fluid balance, edema), there is a lack of reliable bedside tools that specifically inform us of real-time renal venous hemodynamics.

Recently, intrarenal venous flow (IRVF), assessed using renal venous Doppler sonography, has been introduced as a direct visualization of renal venous hemodynamics. Studies outside the critical care field have shown that interrupted, discontinuous IRVF is associated with worsening renal function, even when CVP remains within the normal range [5, 6]. These findings suggest that IRVF is a more sensitive marker of renal congestion than CVP is.

However, only limited research has applied intra-renal venous Doppler sonography in critically ill patients [7, 8], and no study has investigated the utility of IRVF patterns in patients with sepsis after the initial resuscitation phase when the resultant fluid overload was more prominent. Therefore, in this study, we investigated IRVF patterns obtained after the initial resuscitation phase (24 h after sepsis onset) and examined its relationship with CVP in critically ill adult patients with sepsis. In addition, we explored the association between IRVF and subsequent AKI or death. We hypothesized that discontinuous IRVF was associated with elevated CVP and subsequent AKI or death.

## Methods

### Study design and setting

This prospective observational study was conducted at two tertiary care hospitals in Japan. The institutional review board approved this study and waived the requirement for informed consent (Fujita Health University Ethics Review Committee [HM21-442], Okinawa Prefectural Chubu Hospital Research Ethics Committee [2018-129]). Both hospitals had experienced staff intensivists.

### Study participants

We screened all patients admitted to the ICU between August 19, 2019, and July 5, 2020. Patients were eligible if they were adults (≥ 18 years old), were diagnosed with sepsis, stayed in the ICU at least 24 h after the diagnosis of sepsis, had central venous pressure monitored, and received invasive mechanical ventilation. Sepsis was defined based on Sepsis-3, which requires the presence of infection and sequential organ failure assessment (SOFA) scores of ≥ 2 points [9]. We excluded patients who (i) were on maintenance dialysis for chronic renal failure, (ii) were pregnant [10], (iii) had any ureteral obstructions because these affect the IRVF waveforms [11], and (vi) had earlier participation in this study.

### Renal ultrasonography

The main exposure of interest was the IRVF patterns assessed using renal ultrasonography. Before this study, two investigators (KF and IN) underwent the following training sessions for the standardization of renal ultrasonography: they visited the University of Tsukuba Hospital, where the first application study of IRVF in heart failure was conducted and performed renal ultrasonography on 10 patients under the direct supervision of expert sonographers [5]. Next, the study physicians performed renal ultrasonography in 22 ICU patients at each research hospital and received feedback. These sessions were continued until each physician had independent skills.

During the study period, renal ultrasonography was performed at a single time point for each patient to capture renal congestion after sepsis resuscitation. The eligibility criterion (24 h elapsed after the diagnosis of sepsis,) was evaluated daily, and the study physicians performed renal ultrasonography at the bedside in the ICU soon after the patients fulfilled the criterion. Anonymized images and movies of renal ultrasonography were sent to the University of Tsukuba Hospital. The sonographer, blinded to all clinical information, reviewed the images and confirmed the final assessment of the IRVF pattern. Other than the study investigators, no other physician knew the results of renal ultrasonography.

**Fig. 1:**
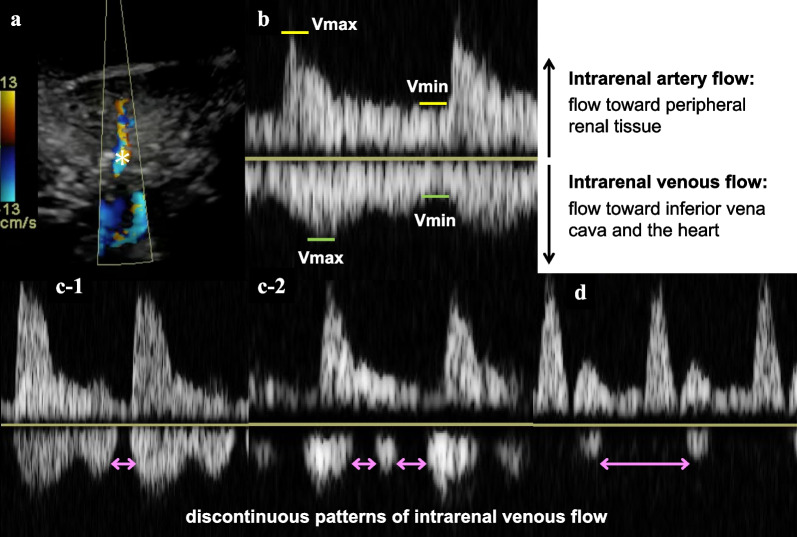
Color Doppler flow images from a right kidney. **a** Doppler sample volume position (*) in the inter-lobar vessels. **b** Intrarenal artery flow (upward Doppler signals), vein flow (downward Doppler signals) and the corresponding maximum and minimum velocity (Vmax, Vmin). The intrarenal venous flow waveforms were classified into a continuous venous flow pattern (**b**) or discontinuous patterns (**c-1**, **c-2**, **d**). Discontinuous patterns include biphasic patterns (**c-1**, **c-2**) and monophasic patterns (**d**) which had at least 1 phase with zero velocity during venous flow in a cardiac cycle as indicated by double-headed horizontal arrows (discontinuous time)

The technical aspects of renal ultrasonography are as follows. Using a sector transducer with a frequency range of 2.5 and 5 MHz [5], we examined the right kidney in the left lateral supine position, except for a patient with postural restrictions. Color Doppler images were used to determine a target, adjacent pair of inter-lobar arteries, and veins with the Doppler velocity range set at approximately 16 cm/s. Inter-lobar arterial and venous pulsed Doppler waveforms were simultaneously recorded (Fig. 1a). The IRVF waveforms (the flow away from the transducer below the baseline) were classified into two waveform patterns: continuous or discontinuous, as defined by Iida et al. [5] (Fig. 1). Specifically, a continuous pattern corresponded to the presence of continuous, uninterrupted venous flow below the baseline throughout the cardiac cycle (Fig. 1b), whereas the discontinuous pattern was a group of patterns that had at least one phase with zero velocity in venous flow during a cardiac cycle (Figs. 1c and d). The venous impedance index (VII), that is, the proportion of reduction in venous flow from its peak velocity (very low [0.2] in healthy subjects), was calculated as the peak maximum flow velocity minus the maximum flow velocity at the nadir in veins divided by the maximum flow velocity [14]. The renal venous stasis index (RVSI), as proposed by Husain et al. [15], was calculated as the index cardiac cycle time minus the renal venous flow time divided by the index cardiac cycle time. For renal arterial flow, renal resistive index (RRI) was calculated as the maximum flow velocity minus the diastolic flow velocity divided by the maximum flow velocity [12]. The RRI in healthy subjects typically ranges between 0.5 and 0.7 [13]. In patients with sinus rhythm, we measured all indices over three cardiac cycles at the end of expiration and averaged them. In patients with atrial fibrillation, we measured the indices at a cardiac cycle where two preceding cardiac cycles had nearly equal durations. Echocardiography was performed at the bedside in the ICU.

### Central venous pressure

The primary measure of systemic congestion was central venous pressure obtained at the time of renal ultrasonography. A single investigator at each study site (KF or IN) measured CVP (a-wave, v-wave, and mean) at the ICU bedside according to the method described by Roger [16] just before or after renal sonography. We measured CVP at the end-expiratory phase in patients in the supine position after carefully zeroing at the mid-thoracic position at the level of the fifth rib. The CVP values were averaged over three cardiac cycles. We recorded the mean CVP of patients with atrial fibrillation. Peripheral edema at baseline was also assessed by palpation of the extremities and the presence of pitting edema.

### Assessment of AKI and outcome measures

The secondary, exploratory outcome measure was a composite of Kidney Disease Improving Global Outcomes (KDIGO) stage 3 AKI [17] including receipt of renal replacement therapy (RRT) or death from any cause within 1 week after IRVF measurement. All patients were followed up until death from any cause, hospital discharge, or the end of the study period (September 14, 2020), whichever came first. We longitudinally assessed the AKI stage for 7 days to evaluate the association between the IRVF pattern and subsequent AKI stage trajectory. Day zero was assigned to the day of IRVF measurement, and the KDIGO stage was recorded from the day before renal sonography (day-1) to day 6. The KDIGO stage classification was based on serum creatinine level or urine output, whichever was worse [17]. If either value was missing, we classified the stage based only on the available data (creatinine or urine output). When both values were missing, we imputed the missing stage with the patient’s last observed KDIGO stage. The baseline value for the serum creatinine criteria was determined by referencing the lowest creatinine value during the previous 12 months before admission. When the pre-admission creatinine level was unavailable, the lowest creatinine value measured within the first 24 h of admission was used as the baseline. The indication, modality, and duration of renal replacement therapy were determined per routine practice at participating hospitals according to the national guidelines [18].

### Sample size estimation

According to a previous study [5], we estimated that the difference between CVP in the continuous IRVF pattern group and discontinuous pattern group was 5 mmHg, and the common standard deviation of CVP in both groups was 5 mmHg. A total of 32 patients were required to confirm the difference between CVP in the IRVF groups with a 2-sided alpha level of 0.05 and power of 80%. Patients were enrolled until the size of both groups reached to at least 16.

### Statistical analyses

For the primary analysis, we examined the association between IRVF pattern (continuous vs discontinuous) and CVP using Student's *t*-test and analysis of covariance (ANCOVA) with adjustment for the APACHE II score. As a secondary exploratory analysis, we performed a generalized estimating equation (GEE) analysis to confirm the association between the IRVF pattern (assessed at a single time point) and the composite of stage 3 AKI or death, assessed repeatedly within 1 week. The motivation for the secondary analysis was as follows: in patients with sepsis, renal congestion that evolved during fluid resuscitation probably deepens the initial worsening of AKI and delays subsequent recovery [19]. Under this hypothesis, and based on a clinical perspective, a method that is sensitive to changes in outcomes over time is desired. GEE is well suited for this purpose as it allows for modelling the correlation between outcomes evaluated at different time points. The APACHE II score, baseline KDIGO stage (on day-1), measurement period, and interaction between measurement period and group (measurement period * group) were included in the GEE model. KDIGO stages were missing in those who experienced rapid recovery from sepsis and survival discharge from the ICU to the ward during the outcome assessment time window (within 1 week after IRVF measurement). Missing values were complemented by the LOCF because the AKI trajectories of these patients were considered stable at the time of missing events. Details of the missing data and a sensitivity analysis without LOCF are presented in Additional file 2: Table S2. Similarly, we performed a GEE analysis to assess the association between baseline CVP and the primary composite outcome within one week. Kaplan–Meier estimation was used to estimate the median survival time for each IRVF group, and the log-rank test was used to assess differences between the groups. As a post hoc supplementary analysis, we added linear regression analysis to assess the potential association between CVP and RVSI, which is a continuous measure, and GEE analysis to assess RVSI and the composite outcomes.

Characteristics are presented using median and interquartile range (IQR) or mean and standard deviation (SD) for continuous variables, and frequencies and proportions for categorical variables. For differences in characteristics between the continuous and discontinuous groups, the Mann–Whitney *U* test was used for continuous variables, and Fisher's exact test was used for categorical variables. Statistical significance was set at a two-sided *p*-value of ≤ 0.05. All analyses were performed using EZR [20] version 1.56 and R version 4.1.1 (R Foundation for Statistical Computing, Vienna, Austria).

## Results

### Characteristics of the study participants

**Fig. 2:**
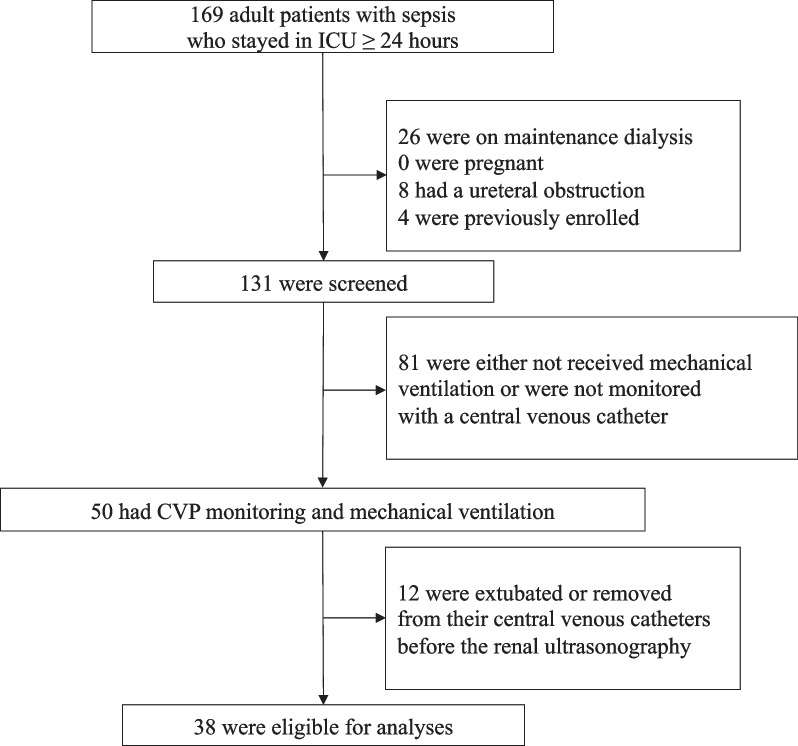
Flow of the study population. CVP denotes central venous pressure

Of 169 adult patients with sepsis who stayed in the ICU for ≥ 24 h during the study period, 50 met the inclusion criteria. Twelve patients were excluded because they were extubated, or their central venous catheters were removed before renal sonography. Finally, 38 patients who met the eligibility criteria were analyzed (Fig. 2). We successfully tracked all patients within the study period until September 14, 2020, when the last patient was discharged. In one patient (1/38 [2.6%]), renal venous Doppler flow was insufficiently detected and substituted with venous flow in the renal pelvis. The patient characteristics are summarized in Table 1. Of the 38 patients, 28 (74%) were male, and the median age was 69.5 (IQR 60–78.8). Twenty-seven patients (71%) were admitted to the ICU from the emergency room. In the entire cohort, 14 patients (36.8%) received RRT for AKI during their ICU stay, six patients (15.8%) died in the ICU, and 12 patients (31.6%) died in the hospital. The median time from ICU admission to renal ultrasonography was 2 days (IQR, 2–3 days; range, 1–13 days). Twenty-two patients (57.9%) had discontinuous intrarenal venous flow pattern. There were 2 monophasic cases and 20 biphasic cases (Fig. 1). The mean of RVSI was 0.20 with a standard deviation of 0.23. Patients with discontinuous patterns were older, had more frequent respiratory infections, and had higher APACHE II scores. Moreover, patients with discontinuous patterns more frequently had a higher cumulative fluid balance and peripheral edema. Creatinine level, AKI stage at the time of renal sonography, and positive end-expiratory pressure (PEEP) were higher in the discontinuous pattern group. Atrial fibrillation was present during renal sonography in three patients in the continuous group and two in the discontinuous group.

**Table 1:** Baseline cohort characteristics, patient information at admission, and renal ultrasonography

	Intrarenal venous flow pattern		*p*-value
	Continuous (*n* = 16)	Discontinuous (*n* = 22)	
Characteristics			
Age (years) (median [IQR])	66.0 [57.0, 71.8]	72.5 [62.0, 81.0]	0.058
Male (%)	13 (81.2)	15 (68.2)	0.469
Body weight (kg) (median [IQR])	67.9 [59.9, 72.3]	55.0 [46.1, 64.2]	0.006
Body mass index (median [IQR])	24.4 [22.9–25.6]	22.5 [19.0–24.2]	0.025
Information at admission			
Primary infectious source, No. (%)			0.016
Respiratory	2 (12.5)	12 (54.5)	
Intraabdominal	5 (31.3)	3 (13.6)	
Urinary tract	0 (0)	1 (4.5)	
Central nervous system	6 (37.5)	1 (4.5)	
Musculoskeletal and skin	1 (6.3)	2 (9.1)	
Other	2 (12.5)	3 (13.6)	
Pre-admission ward, No. (%)			0.788
Emergency department	12 (75.0)	15 (68.2)	
General ward	0 (0)	3 (13.6)	
Operating room	4 (25.0)	3 (13.6)	
Inter-hospital transfer	0 (0)	1 (4.5)	
APACHE II (median [IQR])	20.0 [16.8, 26.0]	25.0 [22.3, 27.0]	0.100
Information at renal ultrasonography			
After ICU admission days (days) (median [IQR])	2.0 [2.0, 2.8]	2.0 [1.3, 3.0]	0.767
Cumulative fluid balance (mL) (median [IQR])	1067 [74, 2199]	2220 [1094, 4540]	0.031
Peripheral edema, No. (%)	6 (37.5)	12 (54.5)	0.342
Serum creatinine (mg/dl) (median [IQR])	1.10 [0.59, 1.59]	1.76 [0.98, 2.96]	0.048
Vital signs			
Mean arterial pressure (mmHg) (median [IQR])	73.5 [70.0, 80.8]	69.5 [61.3, 82.3]	0.399
Heart rate (beats/min) (median [IQR])	71.5 [67.8, 96.0]	76.5 [67.3, 94.8]	0.779
Respiratory rate (median [IQR])	17.5 [16.0, 18.5]	19.0 [17.0, 20.0]	0.188
PaO_2_/FiO_2_ ratio (median [IQR])	374.0 [265.3, 419.3]	309.5 [194.8, 371.0]	0.147
Catecholamines, No. (%)	9 (56.3)	15 (68.2)	0.510
Noradrenaline (mcg/kg/min) (median [IQR])	0.01 [0.00, 0.03]	0.03 [0.00, 0.07]	0.154
Dobutamine, No. (%)	2 (12.5)	3 (13.6)	0.999
Ventilator setting			
Positive end-expiratory pressure (cmH_2_O) (median [IQR])	5.0 [5.0, 6.0]	7.0 [5.0, 9.5]	0.033
Peak inspiratory pressure (cmH_2_O) (median [IQR])	18.5 [16.0, 21.3]	20.0 [17.5, 22.8]	0.313
Minute volume (L/min) (median [IQR])	8.35 [6.92, 10.70]	8.45 [6.56, 9.49]	0.584

*APACHE II score*, acute physiology and chronic health evaluation II score; *bpm*, breath per minute; *FiO*_*2*_, fraction of inspiratory oxygen; *IQR*, interquartile range; *PaO*_*2*_, partial pressure of arterial oxygen

### CVP and other parameters at renal ultrasonography

Table 2 shows CVP and findings of renal ultrasonography and echocardiography. The overall mean central venous pressure was 9.83 mmHg with a mean of 9.24 mmHg (SD: 3.19) in the discontinuous pattern group and 10.65 mmHg (SD: 2.53) in the continuous pattern group. There was no significant difference in CVP between the two groups by Student *t*-test (difference: − 1.4, 95% confidence interval [CI]: − 3.36–0.55, *p* = 0.154) and APACHE2-adjusted ANCOVA (coefficient: − 1.52, 95%CI − 3.59–0.54, *p* = 0.144), and the abnormal IRVF pattern was not associated with CVP (Table 2). We also performed a post hoc supplementary analysis of RVSI, a continuous measure, but the results were similar to those in Table 2, indicating no significant correlation between CVP and RVSI (coefficient: − 2.37, 95% CI − 6.59–1.86, *p* = 0.26). Intrarenal arterial information (renal resistive index) and echocardiographic indices of the left heart and the right heart were similar between the two groups. Patients with discontinuous patterns showed narrower IVC diameters, and their IVCs were more collapsible than those with continuous patterns.

**Table 2:** Findings of central venous pressure, renal ultrasonography, echocardiography at the IRVF measurement

	Intrarenal venous flow pattern		*p*-value
	Continuous (*n* = 16)	Discontinuous (*n* = 22)	
Central venous pressure			
Mean CVP (cmH_2_O) (mean [SD])	10.7 (2.5)	9.2 (3.2)	0.154
CVP a wave (cmH_2_O) (mean [SD])	11.9 (2.7)	11.5 (3.9)	0.771
CVP v wave (cmH_2_O) (mean [SD])	11.4 (3.1)	10.3 (3.6)	0.306
Renal ultrasonography			
Intrarenal venous information			
Venous impedance index	0.41 [0.31, 0.52]	NA	
IRVF discontinuous time^a^ (msec)	NA	251 [123, 312]	
IRVF discontinuous time ratio^b^		NA	0.26 [0.19, 0.45]
Intrarenal arterial information			
Renal resistance index	0.67 [0.61, 0.77]	0.76 [0.71, 0.79]	0.071
Kidney size			
Longer diameter (mm) (median [IQR])	111 [102, 115]	103 [93, 107]	0.051
Shorter diameter (mm) (median [IQR])	55 [51, 63]	51 [46, 58]	0.062
Echocardiography			
Visual ejection fraction, No. (%)			0.587
60% <	7 (43.8)	7 (31.8)	
40–60%	6 (37.5)	12 (54.5)	
20–40%	3 (18.8)	2 (9.1)	
< 20%	0 (0)	1 (4.5)	
E wave (median [IQR])	79.0 [65.8, 87.0]	73.0 [53.6, 102.3]	0.657
A wave (median [IQR])	72.0 [47.3, 88.8]	60.0 [49.3, 83.7]	0.667
Moderate or severe TR, No. (%)	0 (0)	5 (22.7)	0.061
TR-PG (mmHg) (median [IQR])	22.1 [20.0, 25.7]	22.9 [21.0, 32.3]	0.366
Inferior vena cava diameter			
Maximum diameter (mm) (median [IQR])	22 [20, 24]	19 [16, 21]	0.024
Minimum diameter (mm) (median [IQR])	20 [16, 22]	15 [11, 21]	0.029
Caval index^c^ (%)	5.9 [1.0, 17.3]	16.0 [6.6, 30.2]	0.054

*A wave*, Atrial filling wave, *CVP*, central venous pressure, *E wave*, Early diastolic filling wave, *SD*, standard deviation, *IQR*, interquartile range, *IRVF*, intrarenal venous flow, *NA*, not applicable, *TR*, tricuspid regurgitation, *TR-PG*, transtricuspid pressure gradient

^a^IRVF discontinuous time is interruption time of intrarenal venous flow.

^b^Calculated by (IRVF interruption time/cardiac cycle length),

^c^Calculated by [(maximum diameter—minimum diameter)/maximum diameter] × 100

### Patient outcomes

**Fig. 3:**
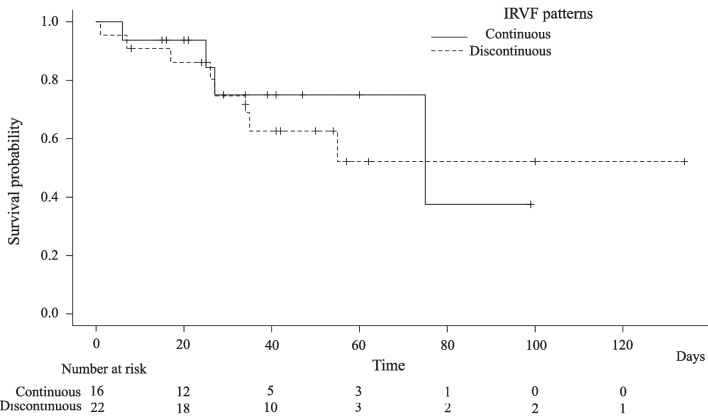
Kaplan–Meier curves for the survival probability according to intrarenal venous flow patterns. Patients were followed from the day before renal ultrasonography until death from any cause or hospital discharge or the end of the study period (September 14, 2020), whichever came first. The vertical tick marks indicate censoring

Table 3 shows patient outcomes. ICU days, ventilator days, hospital days, and death were longer or more frequent in the discontinuous group, although not statistically significant (Table 3). The median survival time was 74 days in the continuous group and could not be calculated in the discontinuous group because the survival probability was never less than 50%. There was no statistical difference between the survival rates in the two groups (*p* = 0.68) (Fig. 3).

**Table 3:** Patient outcomes

	Intrarenal venous flow pattern		*p*-value
	Continuous (*n* = 16)	Discontinuous (*n* = 22)	
ICU days (median [IQR])	5.5 [4.8, 19.0]	9.0 [6.0, 21.0]	0.292
Ventilator days (median [IQR])	4.0 [3.0, 14.5]	7.0 [4.0, 20.5]	0.342
Hospital days (median [IQR])	29.0 [21.0, 42.8]	34.5 [25.0, 57.5]	0.433
ICU death, No. (%)	2 (12.5)	4 (18.2)	0.999
Hospital death, No. (%)	4 (25.0)	8 (36.4)	0.504

*IQR*, interquartile range

### Intrarenal flow patterns and AKI

**Fig. 4:**
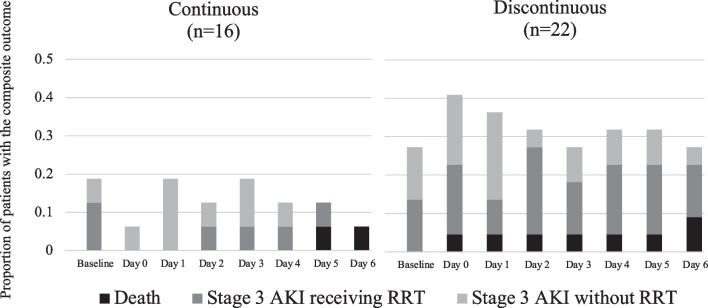
Trajectory of the proportion of patients with the composite outcomes stratified by IRVF patterns and contribution of each component. The composite outcome was the combination of stage 3 acute kidney injury and death. Black bars indicate death, dark gray bars indicate stage 3 AKI patients receiving RRT, and light gray bars indicate stage 3 AKI patients without RRT. Day zero was assigned as the day of IRVF measurement. Those who died during the observation period continued to contribute to the proportions; the denominator was 16 in the continuous group and 22 in the discontinuous group on all days

The trajectory of the proportion of patients who experienced the composite outcome (stage 3 AKI or death) is presented in Fig. 4, stratified by the IRVF pattern. The incidence of the composite outcome tended to be higher in patients with a discontinuous IRVF pattern at all measurement points. Component proportions for the composite outcomes are detailed in Additional file 1: Table S1. Table 4 shows the association between the discontinuous pattern of IRVF with AKI or death. The incidence of events leading to KDIGO stage 3 or death in the one week following IRVF measurement was significantly higher in the discontinuous pattern group (adjusted OR: 9.67, 95% CI 2.13–44.03, *p* = 0.003). The trend was similar in the sub-analysis, with RRT or death as the outcome (Table 4). In contrast, CVP was not significantly associated with the composite outcome (OR: 0.92, 95% CI 0.75–1.13, *p* = 0.439). The results were similar with and without LOCF, confirming the robustness of our conclusions (adjusted OR for the composite outcome in the discontinuous pattern group: 9.92, 95% CI 2.15–45.78, *p* = 0.003) (Additional file 2: Table S2). In our post hoc supplementary analysis, we did not find a significant association between RVSI and the composite outcomes (adjusted odds ratio for the composite outcome in the discontinuous pattern group: 2.25, 95% CI 0.25–20.73, *p* = 0.47).

**Table 4:** Association of IRVF with acute kidney injury, renal replacement therapy, or death

Outcome	*n*/*N*	Crude* OR (95%CI) for IRVF pattern	*p*-value	Adjusted** OR (95%CI) for IRVF pattern	*p*-value
KDIGO stage 3 or death	15/38	7.57 (1.27–45.00)	0.026	9.67 (2.13–44.02)	0.003
RRT or death	10/38	14.15 (3.33–60.1)	< 0.001	29.30 (2.20–391.81)	0.011

*CI*, confidence interval, *IRVF*, Intrarenal venous flow, *KDIGO*, Kidney Disease Improving Global Outcomes, *OR*, odds ratio, *RRT*, Renal replacement therapy

*analyzed by Generalized Estimating Equation

**adjusted for baseline KDIGO stage and APACHE II score and analyzed by Generalized Estimating Equation as well. The reference level was continuous pattern of IRVF. Outcome was repeatedly assessed for 7 days after IRVF measurement.

## Discussion

### Key findings

We investigated IRVF waveform patterns obtained after the initial resuscitation phase (24 h after sepsis onset) and its relationship with CVP and clinical outcomes among critically ill adult patients with sepsis. We demonstrated that measurement of IRVF at the bedside was feasible among invasive mechanically ventilated critically ill patients, and approximately 60% of these patients showed a discontinuous pattern of IRVF on day 2 of their ICU stay. We found no association between IRVF waveform patterns and CVP. In contrast, discontinuous patterns of IRVF were associated with stage 3 AKI or death within the next 7 days. Our results suggest that IRVF could capture renal congestion after the initial resuscitation for sepsis better than CVP and has the potential to guide post-resuscitation fluid therapy in sepsis.

### Relationship to previous studies

Fluid overload and congestion have been increasingly recognized as potential causes of organ dysfunction during critical illnesses. The kidney is encapsulated by a tough fibrous capsule, which is susceptible to the harmful effects of fluid overload during resuscitation [21]. Fluid overload after the initial resuscitation of critically ill patients is strongly associated with AKI and mortality in observational studies and randomized controlled trials [1–3]. However, although peripheral edema, cumulative fluid balance, or CVP were associated with AKI in patients with sepsis [22, 23], these markers may not directly reflect renal congestion. Furthermore, CVP is affected by many factors such as thoracic, pericardial, and abdominal pressures, making its interpretation more complex in the ICU setting [24].

Renal venous Doppler sonography directly visualizes and captures changes in IRVF patterns during renal congestion [6]. During renal congestion, intrarenal venous compliance decreases secondary to elevated renal interstitial pressure [25] or intravascular volume expansion [21], resulting in pulsatile, intermittently interrupted intrarenal venous flow (discontinuous patterns). Discontinuous IRVF patterns were associated with elevated CVP and predicted worsening renal function, hospitalization for heart failure, and death in patients with heart failure [5]. Furthermore, even when CVP remained within the normal range, discontinuous patterns were observed and associated with worse clinical outcomes [5]. These findings suggest that in patients with congestive heart failure, IRVF is a more sensitive marker of renal congestion than CVP. However, the findings in patients with congestive heart failure are not directly applicable to those with sepsis because the two patient groups have different hemodynamics.

Currently, only one study has evaluated the association between IRVF patterns and renal function, including sepsis, in an ICU setting. Spiegel et al. evaluated the IRVF waveform within 24 h of ICU admission among general ICU patients and found no association with major adverse kidney events at 30 days (OR for major adverse kidney events: 2.29, 95% CI 0.69–7.58) [7]. However, renal Doppler was performed in the early phase of critical illness (within 24 h of ICU admission), and the median amount of IV fluid administered at the time of measurement was only 636 mL, which was far less than the typical degree of fluid accumulation during sepsis [7, 26].

In the present study, we enrolled patients with sepsis who were at high risk for fluid overload and renal congestion and focused on the post-resuscitation phase of sepsis (24 h after sepsis onset). The median cumulative fluid balance was 1740 mL on renal Doppler measurements. The design of our study enabled us to evaluate the utility of IRVF in assessing renal venous congestion after the initial resuscitation of sepsis.

### Clinical implications

The absence of an association between CVP and IRVF may highlight distinct hemodynamic features of renal congestion in sepsis, which are different from those in heart failure [27]. The release of inflammatory cytokines and glycocalyx destruction causes peripheral vasodilation and increases vascular permeability in sepsis [28]. As a result, the administered fluid does not remain in the intravascular space and interstitial edema such as renal congestion is not necessarily coupled with elevated central venous pressure. Indeed, discontinuous IRVF was not associated with CVP, but was associated with cumulative fluid balance and subsequent AKI in our study (cumulative fluid balance: discontinuous pattern, 2220 mL; continuous pattern, 1067 mL; *p* = 0.031). These results were consistent with previous findings, showing that CVP had no meaningful relationship with administered fluid volume in septic shock [29]. Our study suggests that the IRVF pattern has the potential to capture organ-specific congestion in the kidney among patients with sepsis, which CVP cannot detect [30]. In our post hoc analysis, we found no significant correlation between RVSI and composite outcomes. Although we treated RVSI as a continuous measure of IRVF, converting IRVF into a continuous variable might be difficult. A recent study by the authors revealed that IRVF patterns arose from a complex combination of renal congestion and right-sided heart hemodynamics (e.g., right ventricular filling abnormality, significant tricuspid regurgitation, and atrial fibrillation) [27]. The waveform or timing of interruptions is important to understand the underlying mechanism of interruption. Therefore, the duration or proportion of interruption (referring to RVSI) alone does not fully represent the pathology and is not necessarily proportional to the severity of renal venous congestion. Indeed, in our analyses, the composite outcomes were seen in discontinuous patterns with a short interruption time (low RVSI) and also in discontinuous patterns with a long interruption time (high RVSI), suggesting a nonlinear association. We suspected that this was the reason why the analysis using RVSI could not find a meaningful association. Thus, we believe that the IRVF pattern itself is a suitable measure of renal venous hemodynamics.

### Strengths and limitations

Our study has several strengths. We demonstrated that the measurement of IRVF at the ICU bedside was feasible for physicians in invasive mechanically ventilated patients. The proportion of inadequate image quality was 2.6% [1/38], which was greatly improved from that in a previous study (inadequate image quality in 25.4% of patients) [7]. In addition, an expert sonographer who was blinded to all clinical information independently confirmed the final assessment of the IRVF pattern. The prospective nature of our study enabled us to carefully measure CVP at the bedside using a standard protocol [16].

Our study has several limitations. First, the analysis of the association of IRVF with AKI or death was exploratory, and the CI of the OR was wide due to the small number of patients. Second, renal ultrasonography was performed by physicians who were aware of the patients’ clinical information. However, we attempted to minimize bias from awareness of clinical information by adopting a blinded assessment of the IRVF pattern by an independent expert sonographer. Third, there exists between-group differences in several key prognostic factors, including age, APACHE score, and primary infectious sources (Table 1). These factors may increase mortality independent of renal congestion or AKI in the Discontinuous group and may explain the observed association. However, the major difference in the composite outcomes between the Continuous and Discontinuous groups arose from increased stage 3 AKI in the Discontinuous group (Fig. 4). The results support our notion that IRVF is not merely a marker of the severity of illness in general but rather a sensitive measure of renal congestion associated with subsequent AKI. Fourth, because we adopted repeating outcome measures, the analytical method used for the association between IRVF and the composite outcome could have led to a decrease in the *p*-values. Although our choice of method (GEE analyses) was intended to capture the AKI trajectory that changes over time after sepsis resuscitation, individuals who died continued to contribute to the analysis after death and may have been overrepresented, resulting in the potential unintentional decrease in the *p*-values. However, as we demonstrated that the proportion of death was low in both groups and the major difference in the composite outcomes between the groups arose from increased stage 3 AKI in the Discontinuous group in Additional file 1: Table S1, we believe that the possibility of an unintentional decrease in the *p*-value due to the mortality difference is unlikely. Fifth, differences in the administered catecholamines or intrathoracic pressure other than fluid overload might affect the IRVF waveform and explain the observed association of IRVF with AKI or death. Although we incorporated baseline kidney function and severity scores into the model to balance the two IRVF groups, a larger study with sufficient adjustment for important factors would be required to confirm our observations.

### Unanswered questions and future research

A previous study showed that blunting of renal venous flow was related to a lower diuretic response, independent of underlying renal function [31]. A discontinuous IRVF pattern may indicate impaired handling of renal fluid. Indeed, in heart failure patients undergoing repeated IRVF measurements, the persistence of a discontinuous IRVF pattern is associated with renal dysfunction progression [6]. Future studies should investigate whether repeated IRVF measurements and IRVF-guided fluid therapy, particularly guided fluid discontinuation and removal, improve AKI and patient outcomes in the ICU setting. In addition, we selected patients who had a central venous catheter and received invasive mechanical ventilation, because we focused on a population with a high risk of renal congestion. Future studies should include a broader range of patients with sepsis.

We believe that with appropriate training, critical care physicians can perform renal sonography at the bedside. Based on our experiences, the key challenges of renal ultrasonography include identifying a suitable probe position and angle for visualizing the interlobar renal veins and setting the Doppler velocity in the lower range appropriate for detecting low-speed venous flow (such as 16 cm/s). Increasing the opportunity for side-by-side guidance and visual aids would expand the implementation of renal ultrasonography in the ICU.

## Conclusions

The IRVF pattern assessed after the initial resuscitation phase (24 h after sepsis onset) was not associated with central venous pressure but was associated with subsequent AKI or death among critically ill adult patients with sepsis. IRVF may have the potential to capture renal congestion at the bedside. Further studies are needed to evaluate whether IRVF-guided fluid discontinuation and removal during the post-resuscitation phase of sepsis improves patient outcomes.

### Supplementary Information

**Additional file 1**: **Table S1**. Component proportions for the composite outcomes.

**Additional file 2**: **Table S2**. Association of IRVF with acute kidney injury, renal replacement therapy, or death without LOCF as a sensitivity analysis.

## Data Availability

The data supporting that support the findings of this study are available from Fujita Health University Hospital and Okinawa Chubu Hospital but restrictions apply to the availability of these data, which were used under license for the current study, and so are not publicly available. Data are, however, available from the authors upon reasonable request and with the institution's permission.

## Abbreviations

AKI: Acute kidney injury
ANCOVA: Analysis of covariance
APACHE II score: Acute physiology and chronic health evaluation II score
CI: Confidence interval
CVP: Central venous pressure
GEE: Generalized estimating equation
ICU: Intensive care unit
IQR: Interquartile range
IV: Intravenous
IVC: Inferior vena cava
IRVF: Intrarenal venous flow
KDIGO: Kidney disease improving global outcomes
LOCF: Last observation carried forward
OR: Odds ratio
RRI: Renal resistive index
RRT: Renal replacement therapy
RVSI: Renal venous stasis index
SD: Standard deviation
SOFA: Sequential organ failure assessment
VII: Venous impedance index
