# Supplementary material for: Association between intrarenal venous flow from Doppler ultrasonography and acute kidney injury in patients with sepsis in critical care: a prospective, exploratory observational study
Source: Crit Care. 2023 Jul 10;27:278. doi: 10.1186/s13054-023-04557-9 (PMC10332034; doi:10.1186/s13054-023-04557-9)
Supplement: Supplementary file 1 — Additional file 1: Table S1. Component proportions for the composite outcomes. [file 13054_2023_4557_MOESM1_ESM.docx]

| Supplementary Table 1. Component proportions (%) for the composite outcomes. | | | | | | |
| --- | --- | --- | --- | --- | --- | --- |
|  | Continuous (n=16) | | | Discontinuous (n=22) | | |
|  | Stage3 AKI (with and without RRT) | Stage3 AKI receiving RRT | Death | Stage3 AKI (with and without RRT) | Stage3 AKI receiving RRT | Death |
| Baseline, No. (%) | 3 (19) | 2 (13) | 0 (0) | 7 (32) | 3 (14) | 0 (0) |
| Day0, No. (%) | 2 (13) | 0 (0) | 0 (0) | 8 (36) | 4 (18) | 1 (5) |
| Day1, No. (%) | 3 (19) | 0 (0) | 0 (0) | 8 (36) | 2 (9) | 1 (5) |
| Day2, No. (%) | 2 (13) | 1 (6) | 0 (0) | 7 (32) | 5 (23) | 1 (5) |
| Day3, No. (%) | 3 (19) | 1 (6) | 0 (0) | 6 (27) | 3 (14) | 1 (5) |
| Day4, No. (%) | 2 (13) | 1 (6) | 0 (0) | 6 (27) | 4 (18) | 1 (5) |
| Day5, No. (%) | 1 (6) | 1 (6) | 1 (6) | 6 (27) | 4 (18) | 1 (5) |
| Day6, No. (%) | 0 (0) | 0 (0) | 1 (6) | 3 (14) | 3 (14) | 2 (9) |
| AKI= acute kidney injury, RRT= Renal replacement therapy | | | | | | |
