# Supplementary material for: Association between intrarenal venous flow from Doppler ultrasonography and acute kidney injury in patients with sepsis in critical care: a prospective, exploratory observational study
Source: Crit Care. 2023 Jul 10;27:278. doi: 10.1186/s13054-023-04557-9 (PMC10332034; doi:10.1186/s13054-023-04557-9)
Supplement: Supplementary file 2 — Additional file 2: Table S2. Association of IRVF with acute kidney injury, renal replacement therapy, or death without LOCF as a sensitivity analysis. [file 13054_2023_4557_MOESM2_ESM.docx]

| Supplementary Table 2. Association of IRVF with acute kidney injury, renal replacement therapy, or death without LOCF as a sensitivity analysis. | | | | | |
| --- | --- | --- | --- | --- | --- |
| Outcome | n / N | Crude* OR (95%CI) for IRVF pattern | p-value | Adjusted** OR (95%CI) for IRVF pattern | p-value |
| KDIGO stage 3 or death | 15/38 | 7.86 (1.28–48.28) | 0.026 | 9.92 (2.15–45.78) | 0.003 |
| RRT or death | 10/38 | 14.71 (3.05–70.89) | 0.001 | 28.41 (2.04–395.24) | 0.013 |
| *analyzed by Generalized Estimating Equation. **adjusted for baseline KDIGO stage and | | | | | |
| APACHE II score and analyzed by Generalized Estimating Equation as well. The reference level | | | | | |
| was continuous pattern of IRVF. Outcome was repeatedly assessed for 7 days after IRVF | | | | | |
| measurement. CI= confidence interval, IRVF= Intrarenal venous flow, KDIGO= Kidney Disease | | | | | |
| Improving Global Outcomes, LOCF= last observation carried forward, OR= odds ratio, | | | | | |
| RRT= Renal replacement therapy | | | | | |
| **Details of missing** | | | | | |
| There existed the two mechanisms of missing in the composite outcomes (stage 3 AKI or death) | | | | | |
| during the time window of the outcome assessment (within 1 week after IRVF measurement): 1) | | | | | |
| missing due to survival discharge from the ICU to the ward; 2) missing due to a lack of SCr and | | | | | |
| hourly UO measurement because patients’ vital signs were rapidly improved and were under less | | | | | |
| intensive monitoring during ICU stay. The AKI trajectories of these patients are considered | | | | | |
| stabilized at the time of missing happens, therefore complementing the missing AKI stages with the | | | | | |
| last observed AKI stage of the patient is reasonable. We employed the last observation carried | | | | | |
| forward (LOCF) to impute missing AKI stages and proceeded to the main analysis. Indeed, missing | | | | | |
| due to survival discharge were observed in 14 patients and their last observed AKI stage was no | | | | | |
| AKI in 13 patients and stage 1 AKI in 1 patient. Missing due to a lack of SCr and hourly UO | | | | | |
| during ICU stay was observed in 2 patients and they had no AKI at the time of missing happened | | | | | |
| and discharged from ICU within 2 days after missing happened. These missing patterns justified | | | | | |
| our choice of LOCF. As a sensitivity analysis, we conducted the analysis without LOCF. The | | | | | |
| results were similar with and without LOCF, confirming the robustness of our conclusions. | | | | | |
